# Editorial to the Special Issue “Homeostasis: Metals and Cellular Redox and Immunity Status”

**DOI:** 10.3390/ijms24054889

**Published:** 2023-03-03

**Authors:** Bernhard Michalke, Vivek Venkataramani

**Affiliations:** 1 Research Unit Analytical BioGeoChemistry, Helmholtz Zentrum München—German Research Center for Environmental Health GmbH, 85764 Neuherberg, Germany; 2 Comprehensive Cancer Center Mainfranken, University Hospital Würzburg, 97080 Würzburg, Germany; 3 Institute of Pathology, University Medical Center Göttingen (UMG), 37075 Göttingen, Germany

It is with great pleasure that we introduce this Special Issue on “Homeostasis: Metals and Cellular Redox and Immunity Status”. The aim of this issue is to explore the intricate relationships between metal homeostasis, cellular redox balance, and immune function.

The importance of maintaining proper metal homeostasis and cellular redox balance cannot be overstated. Metals play a critical role in many biological processes, including enzymatic reactions, signal transduction, and DNA replication. However, an imbalance in metal concentrations can lead to cellular damage and dysfunction. Similarly, the balance of oxidants and antioxidants is crucial for cellular health. Too many oxidants can lead to oxidative stress, while an excess of antioxidants can disrupt signaling pathways. Importantly, both metal homeostasis and cellular redox balance are closely linked to immune function. Metal ions play important roles in immune cell signaling and differentiation, while cellular redox balance affects immune cell activation and proliferation. Disruptions in metal homeostasis and cellular redox balance can lead to impaired immune function and increased susceptibility to infections. The five articles in this collection report on the molecular mechanisms of interfered homeostasis during the pathogenesis of severe diseases.

The first article, “Synaptic Activity Boosts Neuronal Bioenergetics via Iron Metabolism” by Tena Morraja et al. [1] reveals that synaptic activity triggers a transcriptional upregulation of iron metabolism genes, leading to enhanced cellular and mitochondrial iron uptake. This increase in iron availability fuels the electron transport chain complexes, facilitating long-lasting improvements in mitochondrial bioenergetics. In fact, when mitochondrial iron transporter Mfrn1 is inhibited, the activity-mediated bioenergetics boost is blocked. To better understand the lasting effects of synaptic activity on neuronal metabolism, they explored changes to mitochondrial energetics in stimulated neurons. The results showed increased mitochondrial membrane potential and oxygen consumption, with Mfrn1 expression found to be regulated by CREB, a key regulator of synaptic plasticity. This suggests that the expression of synaptic plasticity programs is coordinated with those required to meet the associated increase in energetic demands.

The second manuscript, “Differences and Interactions in Placental Manganese and Iron Transfer across an In Vitro Model of Human Villous Trophoblasts” by Michaelis et al. [2] investigates the transfer of manganese (Mn) and iron (Fe) across a BeWo b30 trophoblast layer. These elements play a crucial role in fetal development, however excess intrauterine Mn is linked to adverse pregnancy outcomes. The study reveals distinct differences in the placental transfer of Mn and Fe, with Mn transfer being largely independent of the applied doses. Concurrent exposure of both elements shows that they share common transfer mechanisms. The authors suggest that Mn transfer likely involves a combination of active and passive transport processes, as DMT1, TfR, or FPN were only marginally altered in BeWo cells despite different exposure scenarios.

Iron is a crucial element in energy metabolism, but when the Fe^2+^/Fe^3+^ ratio goes awry, it can have adverse effects. The third article by Reinert et al. [3] explore safe iron handling. In “High Iron and Iron Household Protein Contents in Perineuronal Net-Ensheathed Neurons Ensure Energy Metabolism with Safe Iron Handling”, the authors test their hypothesis that PN-ensheathed neurons have increased intracellular iron concentration and iron protein levels. They analyze iron and iron proteins in various regions of Wistar rat brains, including the parietal cortex (PC), subiculum (SUB), red nucleus (RN), and substantia nigra (SNpr/SNpc). Their findings show that PN+ neurons have higher iron concentrations than neurons without a PN, along with increased contents of intracellular Tf, TfR, and MTP1. These properties contribute to the low vulnerability of PN+ neurons against iron-induced oxidative stress and degeneration. Reinert et al.’s study highlights the outstanding iron handling capabilities of PN+ neurons, identifying them as a resilient subpopulation of pacemaker neurons.

In the fourth paper, “The Oncopig as an Emerging Model to Investigate Copper Regulation in Cancer”, Carlson et al. [4] investigated the role of copper (Cu), another crucial transition metal with redox capabilities, in cancer. With evidence linking copper to the development of human pathologies, including cancer, the authors sought to utilize animal models that more closely resemble humans in terms of genetics, anatomy, organ size, and disease manifestation. In their review, the authors discuss how the oncopig model can aid in understanding the mechanisms and causal relationships between Cu and molecular targets in cancer. The authors present a transcriptomic analysis revealing significant changes in copper-related molecular pathways in the oncopig model, highlighting its potential as a valuable tool in cancer research.

This Special Issue collection is rounded off by Co-Editor Bernhard Michalke with a review that explores the combination of Inductively Coupled Plasma-Mass Spectrometry (ICP-MS) with advanced sample introduction techniques for elucidating trace element species in pathologic conditions on a molecular level [5]. By combining ICP-MS with different separation or spatial mapping techniques, researchers can gain valuable insights into specific forms of element binding, redox states, and spatial distribution. This review highlights various exciting combinations of sample delivery systems and ICP-MS, as well as natural and artificial tags for drug monitoring. The article also describes quantification methods for Fe^2+^, Fe^3+^, and ferritin-bound iron, as well as for free Cu vs. exchangeable copper, and outlines single-cell ICP-MS for understanding cell-to-cell variance and drug uptake.

We hope that this Special Issue will provide a comprehensive overview of the current state of research on the relationship between metal homeostasis, cellular redox balance, and immunity. We would like to express our gratitude to the authors for their contributions and to the reviewers for their valuable feedback.

Sincerely,

Bernhard Michalke and Vivek Venkataramani.
